# Associations between general and specific psychopathology factors in parents and psychiatric, behavioral, and psychosocial outcomes in offspring: a Swedish population-based register study

**DOI:** 10.1192/j.eurpsy.2024.473

**Published:** 2024-08-27

**Authors:** M. Zhou, H. Larsson, B. D’Onofrio, M. Landén, P. Lichtenstein, E. Pettersson

**Affiliations:** ^1^Karolinska Institutet, Stockholm, Sweden

## Abstract

**Introduction:**

Psychiatric conditions in parents are associated not only with the same condition in offspring, but also with virtually all other psychiatric conditions. However, it remains unknown whether this intergenerational transmission of psychiatric conditions was attributable to broader psychopathology comorbidity or to specific conditions.

**Objectives:**

To estimate associations between general and specific factors of psychopathology in parents, and a wide range of register-based outcomes in their offspring.

**Methods:**

Based on Swedish national registers, we linked 2 947 703 individuals born in Sweden between 1970 and 2000 to their biological parents (1 705 780 pairs of parents) and followed them to December 31, 2013. First, we estimated one general and three unrelated (specific) psychopathology factors (capturing internalizing, externalizing, and psychotic problems, respectively, independently of general psychopathology) based on nine parental register-based psychiatric diagnoses and violent criminal court convictions. Second, we regressed each offspring outcome on the latent general and three specific factors simultaneously.

**Results:**

The general psychopathology factor in parents was significantly associated with all 31 offspring outcomes (mean Odds Ratio (OR) = 1.22; range: 1.08–1.40), which means that children whose parents scored one standard deviation above the mean on general psychopathology had, on average, a 23% higher probability of all outcomes. The specific psychotic factor in parents was primarily associated with psychotic-like outcomes (mean OR = 1.17; range: 1.05–1.25), and the specific internalizing factor in parents was primarily associated with offspring internalizing (mean OR = 1.11; range: 1.11–1.13) and neurodevelopmental outcomes (mean OR = 1.07; range: 1.02–1.10). The specific externalizing factor in parents was associated with externalizing (mean OR = 1.27; range: 1.21–1.32) and internalizing outcomes (mean OR = 1.10; range: 1.01–1.13).

**Conclusions:**

The intergenerational transmission of psychiatric conditions across different types of spectra appeared largely attributable to a parental general factor of psychopathology, whereas specific factors were primarily responsible for within-spectrum associations between parents and their offspring. Service providers (e.g., child psychologists, psychiatrists, teachers, and social workers) might benefit from taking the total number of parental mental health problems into account, regardless of type, when forecasting child mental health and social functions.

**Disclosure of Interest:**

None Declared

